# Diatom and pollen atlas dataset from the Northern Gulf of Mexico, USA

**DOI:** 10.1016/j.dib.2023.109033

**Published:** 2023-03-02

**Authors:** Erika Rodrigues, Kam biu Liu, Paulo Eduardo De Oliveira, Beatriz L Figueiredo, Qiang Yao

**Affiliations:** a Department of Oceanography and Coastal Sciences, Louisiana State University, Baton Rouge, LA 70803, United States of America; b Institute of Geosciences, University of São Paulo, São Paulo, Brazil; c Department of Oceanography and Coastal Sciences and Coastal Studies Institute, Louisiana State University, Baton Rouge, LA 70803, United States of America; d Keller Science Action Center, The Field Museum of Natural History, Chicago, IL 60605, USA; e University of São Paulo, CENA/^14^C Laboratory, Av. Centenário 303, 13400-000 Piracicaba, São Paulo, Brazil

**Keywords:** Palynology, Diatom, Paleoecology, Microfossil, Paleontology

## Abstract

Diatom and pollen references such as atlases and identification keys are remarkably rare from the Gulf Coast region of the United States. This dataset describes modern and fossil diatom and pollen from Galveston Bay, Texas to Cedar Keys Florida, USA. An illustrated and descriptive atlas of diatom and pollen was compiled from original data to facilitate the identification of microfossil in sediments. For diatom atlas, we include light micrographs and detailed descriptions of a total of 32 diatom species, including 9 marine diatom species, 18 estuarine diatom species, and 5 freshwater diatom species. For pollen atlas, we include light micrographs and descriptions of a total of 28 pollen and non-pollen palynomorphs, including 3 mangrove taxa, 12 upland (tree and shrub) taxa, and 10 herbaceous taxa. The diatom atlas is referenced from LSU Global Change and Coastal Paleoecology Laboratory's light micrographs collection. The pollen and diatom datasets are associated with research articles by Yao et al. [1,2].

**Specifications Table**

**Table:** 

Subject	Earth and Planetary Sciences - Paleontology
Specific subject area	Diatom and pollen atlas
Type of data	Image and table
How the data were acquired	The images were acquired via an Olympus Light Microscope with a LC35 camera (3.5-megapixel CMOS sensor) in conjunction with ZEN Blue 2012 imaging software to photograph pollen grains under 1000x magnification.
Data format	Raw
Description of data collection	Pollen identification was based on microstructural analysis of the aperture and ornamentation, such as the characteristics of pore, colpus, and texture. At least 300 pollen grains were counted for each pollen sample. Diatom identification was based on microstructural analysis of the silicified cell wall, such as pore size, external layer, volume of diatoms, and shapes. At least 500 valves were counted for each diatom sample.
Data source location	• City/Town/Region: Northern Gulf of Mexico, USA • Country: USA • Latitude and longitude (and GPS coordinates, if possible) for collected samples/data: ∼29°10′ to 29°5′ N, ∼94°44′ to 82°6′ W
Data accessibility	Repository name: Mendeley Data Data identification number: DOI: 10.17632/ssdf8grtpf.4 DOI: 10.17632/rzb4t7cf3w.1 Direct URL to data: https://data.mendeley.com/datasets/ssdf8grtpf https://data.mendeley.com/datasets/rzb4t7cf3w
Related research article	Yao, Q., Liu, K.B., Rodrigues, E., Fan, D. and Cohen, M., 2022. A palynological record of mangrove biogeography, coastal geomorphological change, and prehistoric human activities from Cedar Keys, Florida, USA. Science of The Total Environment, p.160189. https://doi.org/10.1016/j.scitotenv.2022.160189

## Value of the Data

• The pollen atlas facilitates the identification of pollen taxa from subtropical coastal environments including mangroves, maritime forests, tidal wetlands, and beaches and dunes.
• The diatom atlas facilitates the identification of diatom species live in marine, estuarine, and freshwater environments.
• The relatively abundance of different diatom species permits the assessment of salinity, water level, and other environmental factors in the open bay water and on the coastal zone.
• The relative abundance of different pollen taxa permits the assessment of vegetation, landform, salinity, and other environmental factors in paleo-ecological research.

## 1 Objective

Our objective is to provide an illustrated and descriptive diatom and pollen atlas from the Northern Gulf of Mexico coast in United States. These data can be used to reveal the dynamics of vegetation and phytoplankton communities in a millennial timescale and document the salinity, water level, and ecological variations associated with the rapid climate change during the Anthropocene.

## 2 Data Description

Diatoms are one of the most productive photosynthesizing algae living in offshore, inshore, and freshwater environments worldwide. Diatom analysis is widely used in paleoenvironmental studies to reveal the salinity, temperature, water depth, and other environmental factors in the past [3]. On the other hand, palynology is a time-tested technique in the reconstruction of paleoenvironmental changes and vegetation dynamics. Data from pollen analysis can reveal the vegetation community, climate, landform, and other environmental conditions during the past decades to millennia.

However, diatom and pollen atlases and identification keys are remarkably rare from the Northern Gulf of Mexico coast, which can hinder paleoecological reconstruction in the region. This dataset describes modern and microfossil diatom and pollen from Bolivar Flats near Galveston Bay, Texas, and from Cedar Keys National Wildlife Refuge, Florida, USA. An illustrated and descriptive atlas of diatom (section 2.1-2.3) and pollen (section 2.4-2.7) was compiled to facilitate the identification of these microfossils in sediments. We include light micrographs and detailed descriptions of a total of 32 diatom genera and species and 28 pollen and non-pollen palynomorphs. The original count and relative abundance of all the pollen [4] and diatom taxa [5] is listed in Mendeley Data. These data can be used as a reference for future studies to conduct pollen and diatom analyses from across the Gulf of Mexico.

### 2.1 Identification key (marine diatom)

**Fig. 1:**
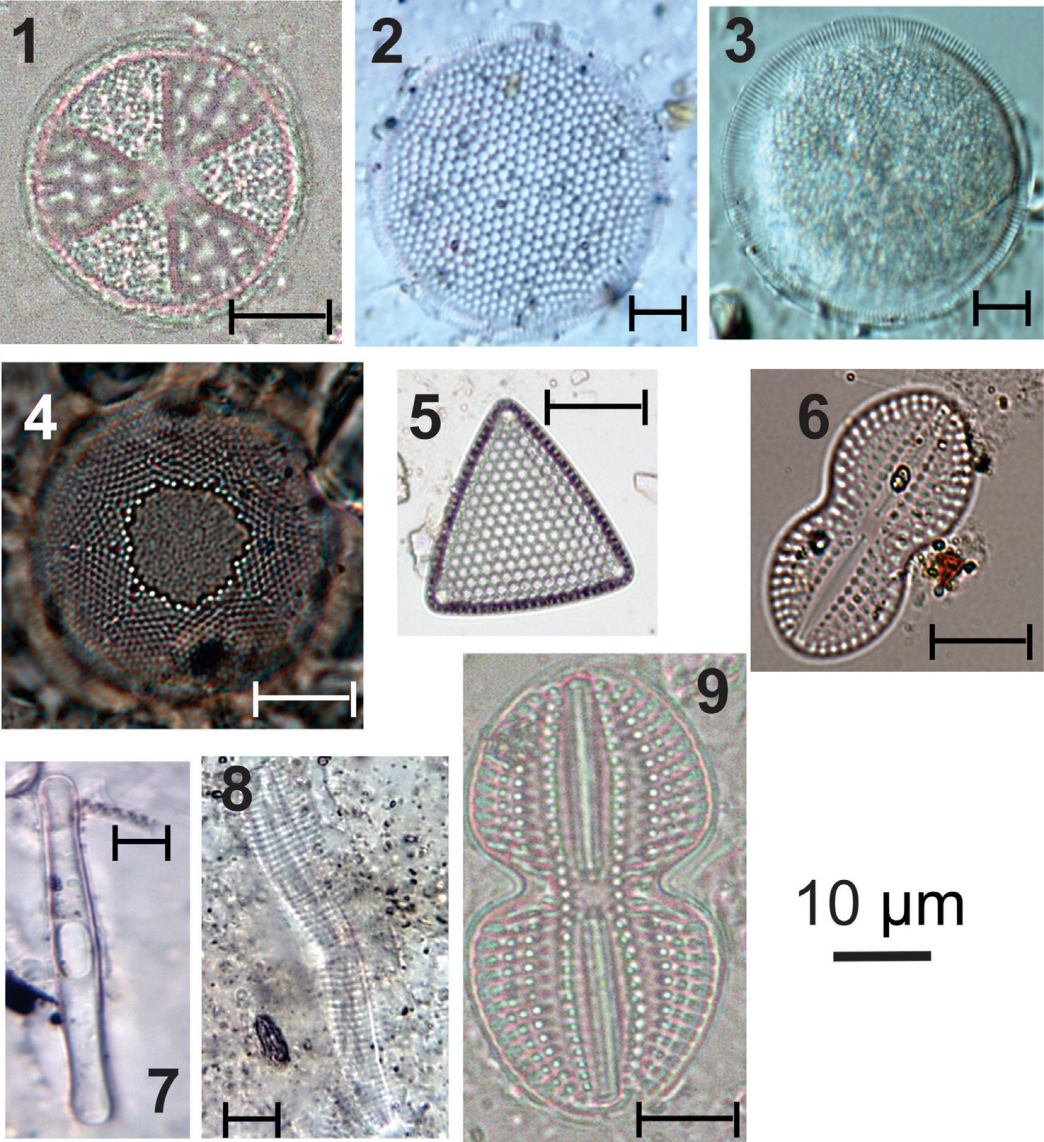
light micrographs of marine diatoms, including: **1**. Actinoptychus senarius, **2**. Coscinodiscus excentricus, **3**. Coscinodiscus oculus-iridis., **4**. Podosira stelliger, **5**. Triceratium favus, **6**. Diploneis sp., **7**. Grammatophora oeanica, **8**. Achnanthes pseudogroenlandica, **9**. Diploneis weissflogii

***Actinoptychus senarius*** (#1 in Fig. 1): Circular valves; wavy valve surface, divided into depressed and elevated sectors; hyaline central area; hexagonal areolas covered by delicate punctuations; radial striations; Marginal rhinoportulas present at the base of each elevated sector [6]. Valve diameter: 17.4-55.3 µm; 4-5 areolas in 10 µm.

***Coscinodiscus excentricus*** (#2 in Fig. 1): Circular valves; flat valve surface; hexagonal areolas arranged in tangential rows; presence of fultoportules distributed over the valve surface and a double row of marginal fultoportulae; an unclear marginal rhinoportula [7]. Valve diameter: 39.5-51.4 µm; 5 areolas in 10 µm; 8 marginals, fultoportulas in 10 µm.

***Coscinodiscus oculus-iridis*** (#3 in Fig. 1): Circular valves; flat valve surface; five central areolas arranged in the rosette form; curved tangential grooves of different sizes; streaks sometimes interrupted by larger areolas; marginal rimoportula (difficult to visualize). Valve diameter: 21- 87 µm; 3-4 areolas in 10 µm [6].

***Podosira stelliger*** (#4 in Fig. 1): Circular valves; convex valve surface; central area contains few tiny areolas arranged irregularly and delimited by an irregular border originated by an uneven shortening of the striae in the peripheral zone; hexagonal areolas forming striations radials, divided into sectors [8]. Valve diameter: 43.5-46.3 µm; 15-16 grooves in 10 µm; 12-14 areolas in 10 µm; 13 sectors.

***Triceratium favus*** (#5 in Fig. 1): Triangular valves with rounded angles; valve surface ornamented with robust hexagonal areolas; arranged in parallel lines; presence of an ocellus in each valve angle [9]. Valve length: 47-61 µm; pervalvar axis: 27-31 µm; side: 71.1-122.5 µm; base: 75.8-118.5 µm; 2 areolas in 10 µm.

***Diploneis* sp.** (#6 in Fig. 1): Linear-elliptic valves; rounded ends; linear and narrow raphe sternum; quadrangular central area; filiform raphe; indistinct longitudinal channel; striations parallel to radiated in ends; delicate areolas that are difficult to count.

Apical axis: 16.6-17.4 µm; transapical axis: 7.9 µm; 12-14 grooves in 10 µm.

***Grammatophora oeanica*** (#7 in Fig. 1): Central region has elliptical and hyaline structures that does not maintain contact with the lateral limit of the valve. Rod-shaped valve with rounded ends before the end of its extension; finely punctated puncta in quincunx; very narrow pseudoraphe; small polar field. Marine taxa such as *Grammatophora oceanica* and *Tryblionella granulata* significantly increased in abundance with the presence of mangroves [10]. Valve diameter: 5 µm. Apical axis 40–75 µm; Transapical axis 4–6 µm. Striae, 21–23 in 10 µm.

***Achnanthes pseudogroenlandica*** (#8 in Fig. 1): Oblong-linear valves, with rounded ends; upper valve with narrow axial area; costate striae, 4-6 in 10 µm; coarsely punctate; crossed by a longitudinal line; lower valve with indistinct axial area and a broad stauros; lower valve with rudimentary diaphragms. Striae: 5-6 in 10 µm, 3-4 coarse puncta.

***Diploneis weissflogii*** (#9 in Fig. 1): Elliptical valves, strongly constricted in the median region and divided into two equal parts; round ends; linear and narrow raphe sternum; quadrangular central area; filiform raphe; indistinct longitudinal channel; radiate striae; coarse areolas [11]. Axle apical: 23.7-31.6 µm; transapical axis: 5.5-8.7 µm; 8-10 grooves in 10 µm; 9 areolas in 10 µm.

### 2.2 Identification key (estuarine diatom)

**Fig. 2:**
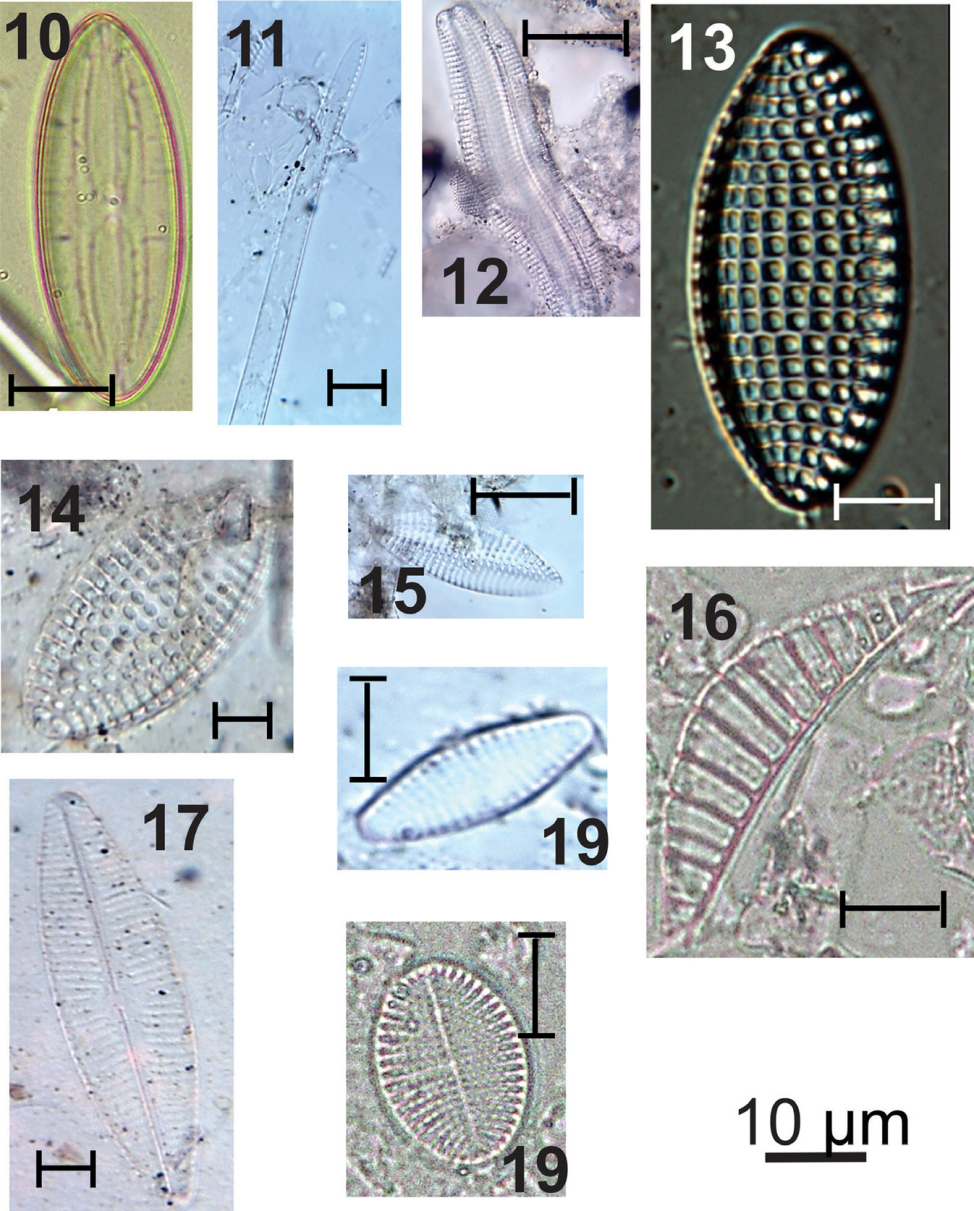
light micrographs of estuarine diatoms, including Light micrographs of estuarine diatoms, including: **10**. Fallacia pygmaea, **11**. Nitzschia obtusa, **12**. Achnanthes inflata, **13**. Tryblionella granulate, **14**. Nitzschia granulata var. hyalina, **15**. Nitzschia granulata var. hyalina, **16**. Rhopalodia gibberula, **17**. Navicula recens, **18**. Desikaneis gessneri, **19**. Cocconeis sp.

***Fallacia pygmaea*** (#10 in Fig. 2): Elliptical valves; rounded ends; arched and narrow raphe; central area joined to an “H”- shaped hyaline structure that interrupts the striae near the raphe; radiated striae; inconspicuous areolas. Apical axis: 14.4 µm; transapical axis: 7.5 µm; 16 grooves in 10 µm.

***Nitzschia obtusa*** (#11 in Fig. 2): Linear-lanceolate sigmoid valves; rostrate-rounded ends; marginal fibulae in the medial region of the valve; parallel striations throughout the valve extension; delicate areolas. Apical axis: 60-93.2 µm; transapical axis: 7.9-8.1 µm; 20-24 grooves in 10 µm; 16-20 areolas in 10 µm; 5-7 fibulas in 10 µm.

***Achnanthes inflata*** (#12 in Fig. 2): Linear valves; rounded to broadly rounded ends; valve with raphe: sternum of raphe central, linear, narrow; radiated striae; rounded areolas. Apical axis: 21.3-47.4 µm; transapical axis: 10.3-15 µm; 11-14 striations in 10 µm; 16 areolas in 10 µm.

***Tryblionella granulate*** (#13 in Fig. 2): Elliptical to elliptical-lanceolate valves; rounded ends; marginal and transversely elongated fibulae; coarsely areolate striations; quadrangular areolas. Apical axis: 22.1-30.8 µm; transapical axis: 9.5-15 µm; 5-7 grooves in 10 µm; 6 areolas in 10 µm; 5-7 fibulas in 10 µm.

***Nitzschia granulata* var. *hyalina*** (#14, 15 in Fig. 2): Elliptical valves; rounded ends; equidistant marginal fibulae; stretch marks interrupted parallels by a longitudinal hyaline area; quadrangular areolas. Apical axis: 20-30 µm; transapical axis: 9-16 µm; 7 grooves in 10 µm; 5 areolas in 10 µm; 7 fibulas in 10 µm.

***Rhopalodia gibberula*** (#16 in Fig. 2): Dorsiventral valves; convex dorsal margin; concave ventral margin; rounded ends; striations and ribs parallel to radiate at extremities; rounded areolas. Apical axis: 36.3-43.5 µm; transapical axis: 10.3-11.9 µm; 12-20 grooves in 10 µm; 10 areolas in 10 µm; 2 ribs in 10 µm.

***Navicula recens*** (#17 in Fig. 2): Linear-lanceolate valves; attenuated-rounded ends; linear raphe-sternum; rounded central area, little expanded; filiform raphe; striae radiate at the extremities, more widely spaced in the middle valve region; inconspicuous areolas. Apical axis: 18.2-36.4 µm; transapical axis: 5.5-7.1 µm; 11-16 grooves in 10 µm.

***Desikaneis gessneri*** (#18 in Fig. 2): Elliptical valves; rhombo-elliptic to rhombo-lanceolate; rounded ends; raphe sternum broad, lanceolate; distinct stature; alternating and radiating striations throughout the valve. Apical axis: 8.7-18.2 µm; transapical axis: 4.7-7.1 µm; 9-12 grooves in 10 µm.

***Cocconeis* sp.** (#19 in Fig. 2): Elliptical valves; rounded ends; raphe valve; rounded central area; filiform raphe; rounded areolas; curved-radiate striations.

Apical axis: 14.1-25.3 µm; transapical axis: 7.9-15 µm; 10-14 striations in 10 µm and 16-20 areolas in 10 µm.

**Fig. 3:**
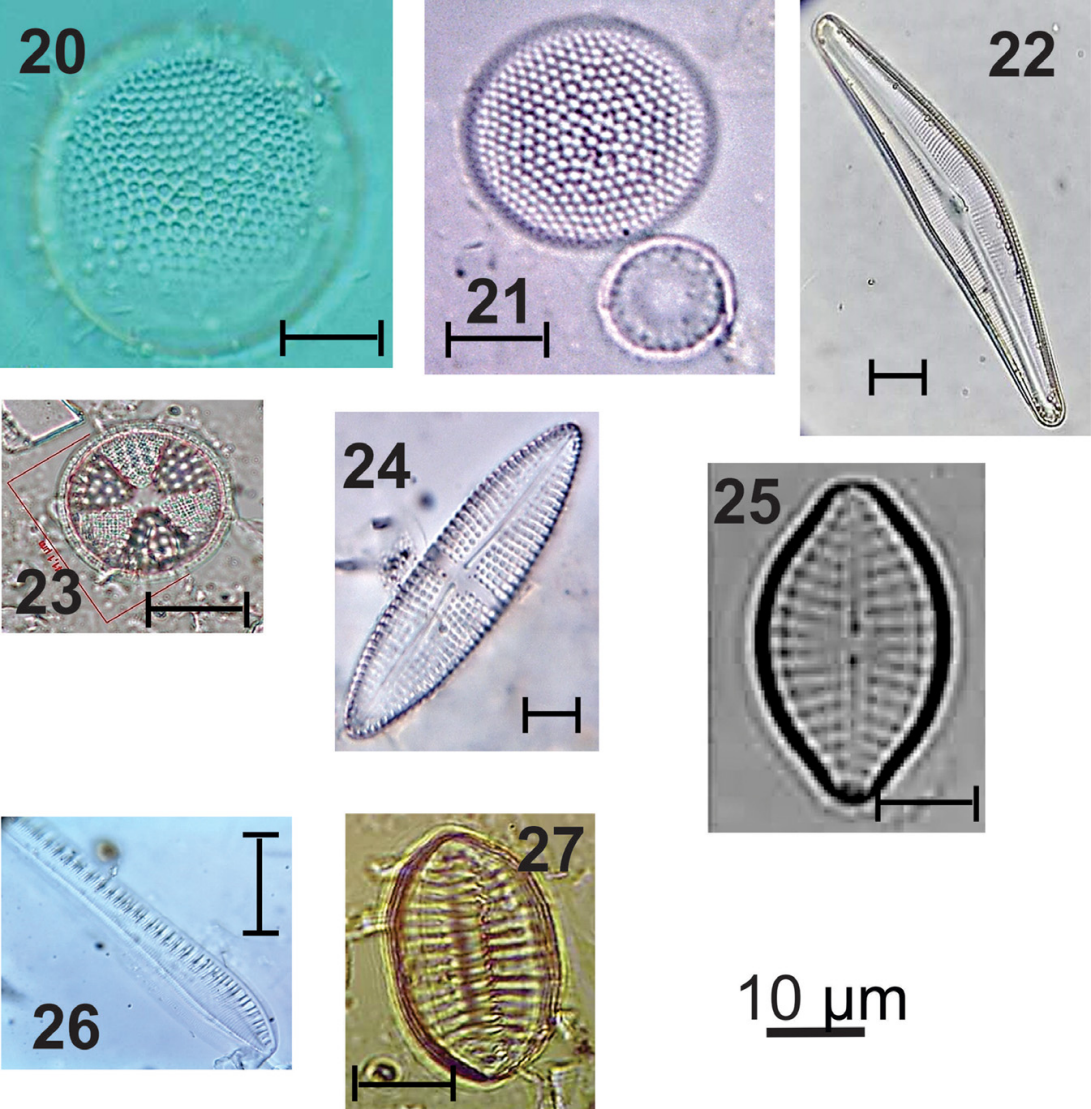
light micrographs of estuarine diatoms, including: **20**. *Thalassiosira eccentrica,***21**. *Thalassiosira tenera*, **22**. *Cymbella lanceolata*, **23**. *Actinoptychus* sp., **24**. *Achnanthes brevipes,***25**. *Planothidium delicatulum,***26**. *Nitzschia* sp., **27**. *Nitzschia* levidensis

***Thalassiosira eccentrica*** (#20 in Fig. 3): Circular valves; flat valve surface; hexagonal areolas arranged in tangential rows; presence of fultoportules distributed over the valve surface. Valve diameter: 39.5-51.4 µm; 5 areolas in 10 µm; 8 marginal fultoportulas in 10 µm.

***Thalassiosira tenera*** (#21 in Fig. 3): Circular valves; flat valve surface; hexagonal areolas arranged in diagonal rows; central areola; central fultoportula. Valve diameter: 7.5 µm; 10-12 areolas in 10 µm; 4 marginal fultoportulas in 10 µm.

***Cymbella lanceolata*** (#22 in Fig. 3): Valves are long and lanceolate with rounded apices. The dorsal margin is moderately arched. The ventral margin is concave with a gibbous center. The axial area is narrow and linear, slightly wider than the raphe. The central area is small and ovoid. The raphe is lateral, becoming filiform near the proximal and distal ends. Terminal raphe fissures are deflected dorsally at an angle of almost 90 degrees. Radiate striae.

Apical axis: 48 µm; transapical axis: 7 µm; 5 dorsal and ventral striations in 10 µm; 24 areolas in 10 µm.

***Actinoptychus* sp.** (#23 in Fig. 3): Valves with little pronounced dorsiventrality; concave dorsal margin; ventral margin convex; attenuated-rounded ends; sternum of the raphe lanceolate on the side dorsal and linear narrow on the ventral side; rounded central area; circular valves; hyaline central area; hexagonal areolas; radial striations; Marginal rhinoportulas present at the base of each elevated sector. Valve diameter: 25 µm; 4 areolas in 10 µm.

***Achnanthes brevipes*** (#24 in Fig. 3): Elliptical valves; rounded ends; raphe valve: central raphe sternum, linear; filiform raphe; radiated striae; areolas coarse, rounded. Apical axis: 28.8-32.5 µm; transapical axis: 7.5-9.4 µm; 8-10 striations in 10 µm; 16 areolas in 10 µm.

***Planothidium delicatulum*** (#25 in Fig. 3): Lanceolate valves; attenuated-rounded ends; raphe valve: raphe sternum linear, narrow; rounded central area; filiform raphe; radiated striae; inconspicuous areolas. Apical axis: 9.5-15.8 µm; transapical axis: 5.5-7.9 µm; 10-12 grooves in 10 µm.

***Nitzschia* sp.** (#26 in Fig. 3): Lanceolate valves; broadly toned-rounded ends; marginal fibulas not equidistant from each other; semicircular hyaline area close to the proximal ends of the raphe; delicate striae; inconspicuous areolas. Apical axis: 34.4 µm; transapical axis: 8.4 µm; 26 grooves in 10 µm; 9 fibulas in 10 µm.

***Nitzschia levidensis*** (#27 in Fig. 3): Linear-lanceolate valves with longitudinal undulation; sub-faced ends; marginal fibulas conspicuous and equidistant from each other; coarse parallel striations; inconspicuous areolas. Apical axis: 22.1- 49.8 µm; transapical axis: 10-24 µm; 4-10 grooves in 10 µm; 7 fibulas in 10 µm.

### 2.3 Identification key (freshwater diatom)

**Fig. 4:**
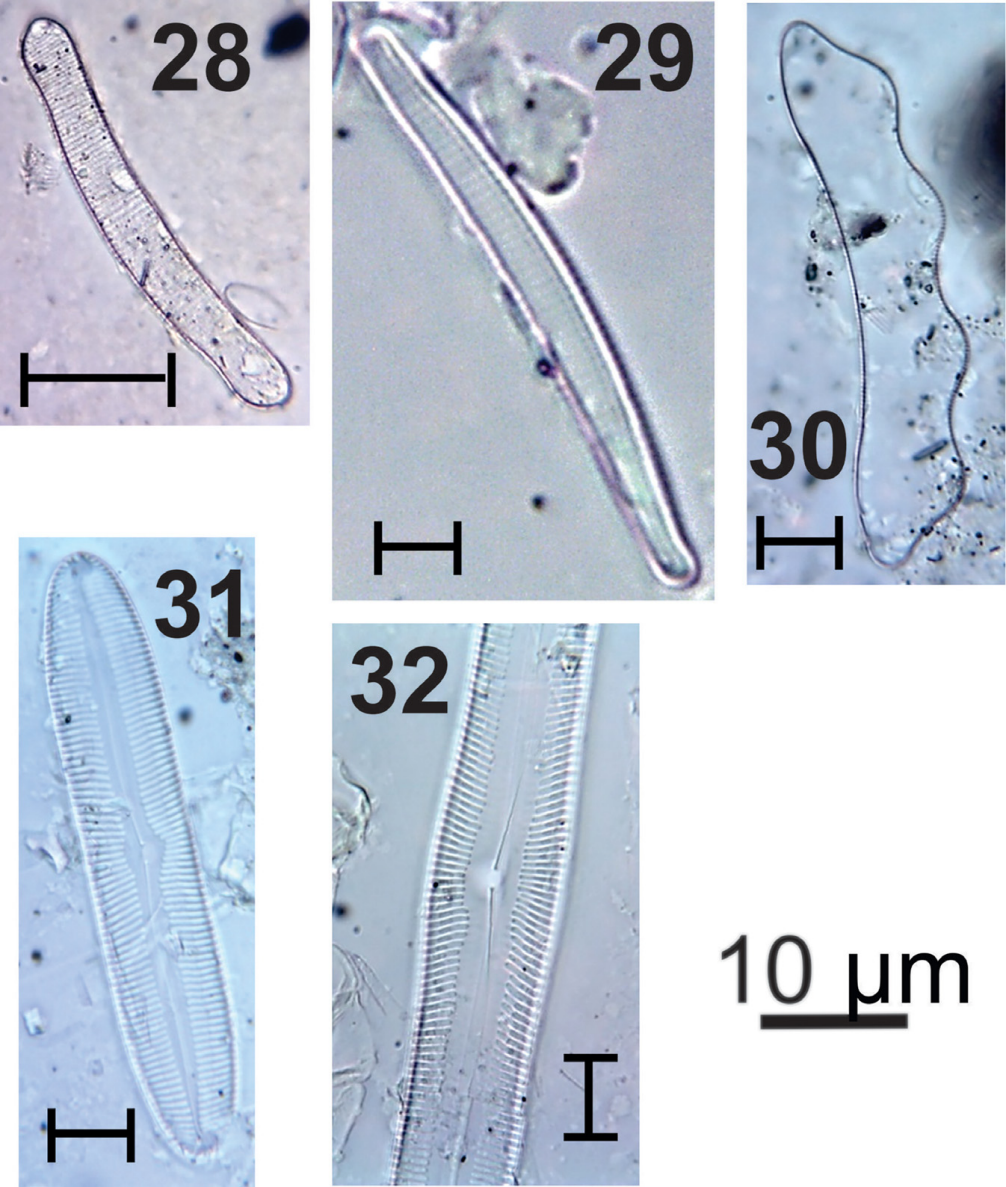
Light micrographs of freshwater diatoms, including: **28**. *Eunotia* sp.1, **29**. *Eunotia* sp.2, **30**. *Eunotia zygodon*, **31**. *Pinnularia macilenta*, **32**. *Pinnularia viridis*.

***Eunotia* spp.** (#28, 29 in Fig. 4): Valves with slightly convex dorsal margin; concave ventral margin; rounded ends; terminal nodules on the extremities; parallel to radiated striations; inconspicuous areolas. Apical axis: 66.3-143.5 µm; transapical axis: 6.3-7.8 µm; 14-20 striations in 10 µm.

***Eunotia zygodont*** (#30 in Fig. 4): Valves with convex dorsal margin showing two humps; concave ventral margin; wedged ends, detached from the valve body; terminal nodules on the extremities; striations parallel to radiate towards to the ends; rounded areolas. Apical axis: 55.3-107.4 µm; transapical axis: 11.9-15.8 µm; 10-12 grooves in 10 µm; 20-24 areolas in 10 µm.

***Pinnularia macilenta*** (#31 in Fig. 4): Linear valves, slightly swollen in the median region; broadly rounded ends; sternum of the linear raphe; widely expanded central area reaching the valve margins; complex raphe; striations radiate to converging towards the ends. Apical axis: 64.2 µm; transapical axis: 8.4 µm; 5 striations by 10 µm.

***Pinnularia viridis*** (#32 in Fig. 4): Linear-elliptic valves; rounded ends; sternum of the linear raphe; rounded central area, more expanded on one side of the valve; complex raphe, proximal ends flexed; central node; striations radiate to converging at the ends of the valve. Apical axis: 123.2-157.2 µm; transapical axis: 10.3-25.3 µm; 9-11 striations in 10 µm.

### 2.4 Identification key (mangroves) [12,13]

**Fig. 5:**
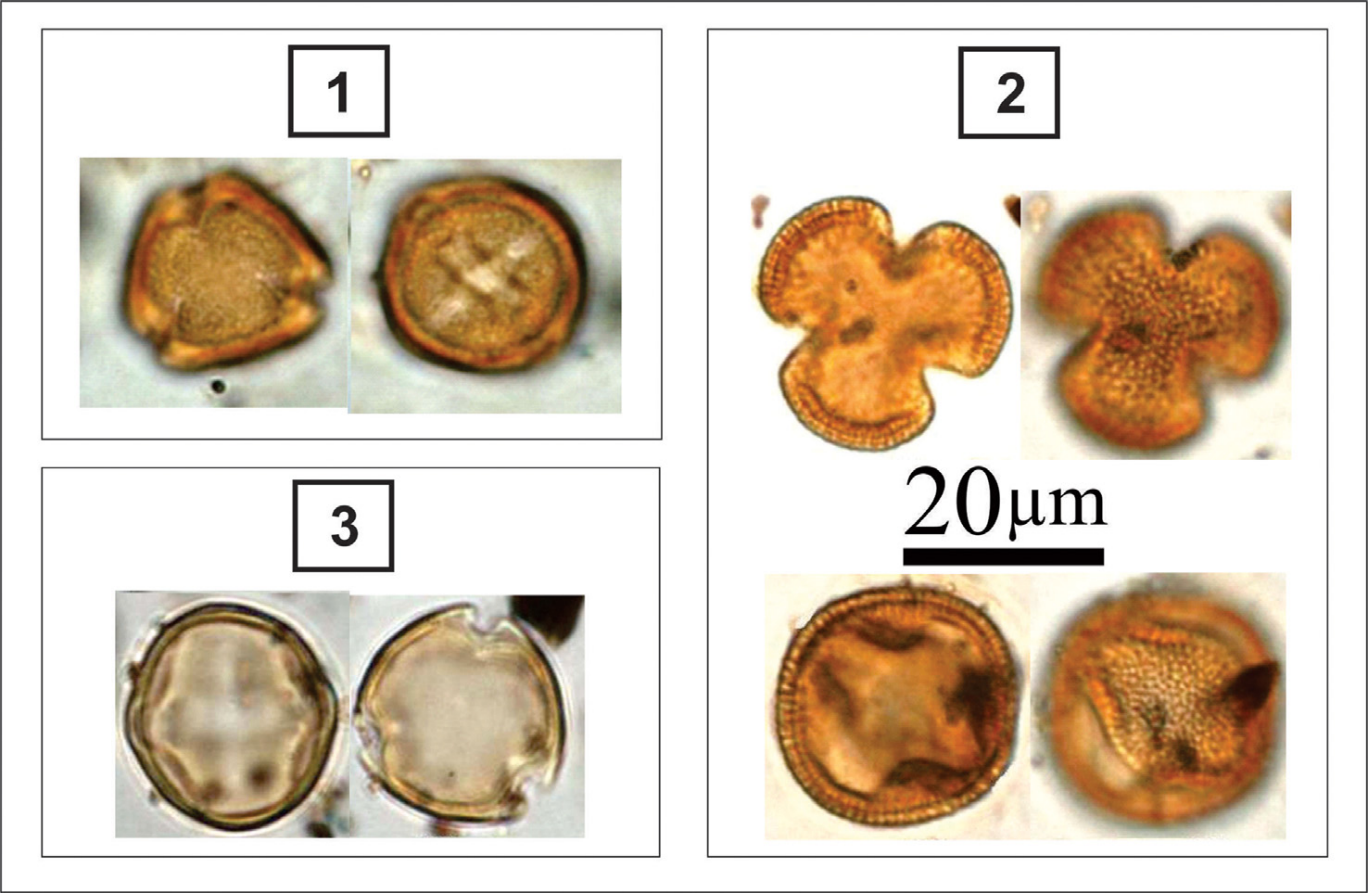
Light micrographs of mangrove taxa, including: **1.***Rhizophora mangle* (Rhizophoraceae); **2.***Avicennia germinans* (Avicenniaceae); **3.***Laguncularia racemosa* (Combretaceae).

***Rhizophora mangle*** (Rhizophoraceae; red mangrove; #1 in Fig. 5): Subspheroidal to prolate grain; tricolporate; finely reticulate; colpus length: 15–20 µm; lalongate pore; polar axis: 22–27 µm; equatorial axis: 17–21 µm

***Avicennia germinans*** (Avicenniaceae; black mangrove; #2 in Fig. 5): Spheroidal grain; tricolporate; reticulate; colpus length: 20–25 µm; elongated pore; polar axis: 32–38 µm; equatorial axis: 25–30 µm

***Laguncularia racemosa*** (Combretaceae; white mangrove; #3 in Fig. 5): Subspheroidal to prolate grain; tricolporate; finely reticulate; colpus length: 17–22 µm; oval pore; polar axis: 22–28 µm; equatorial axis: 15–23 µm

### 2.5 Identification key (tree and shrub taxa)

**Fig. 6:**
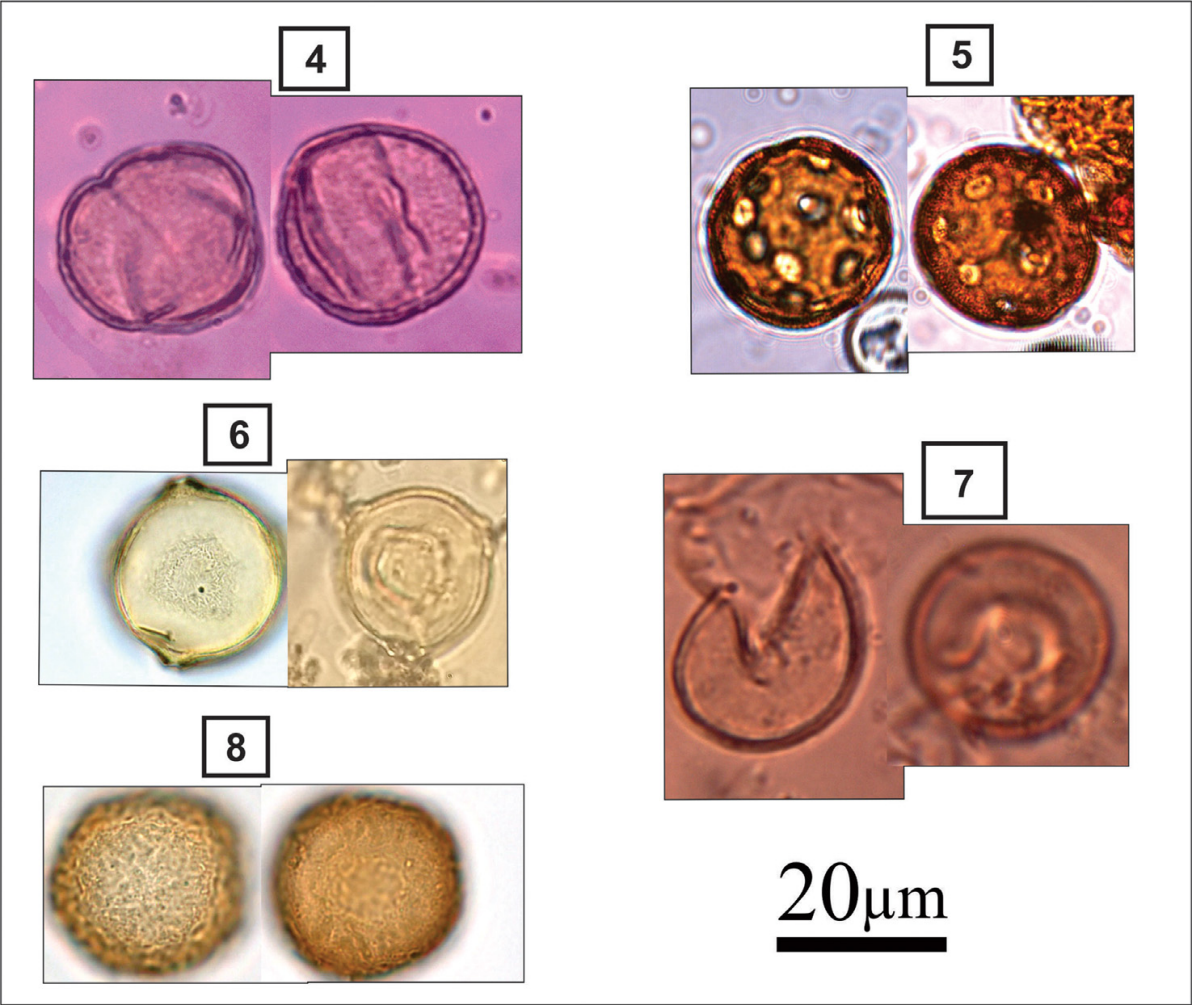
Light micrographs of tree and shrub taxa, including: **4.***Quercus* sp.; **5.***Liquidambar* sp.; **6.***Ostrya* sp.; **7.***Taxodium* sp.; **8.***Ulmus* sp.

***Quercus* sp.** (#4 in Fig. 6): Subspheroidal to prolate grain; tricolpate–tricolporoidate; scabrate; colpus length: 18–24 µm; polar axis: 25–32 µm; equatorial axis: 21–28 µm.

***Liquidambar* sp.** (#5 in Fig. 6): Spheroidal grain; polyporate (12-20 pores); circular amb; circular pore; diameter: 30–35 µm.

***Ostrya* sp.** (#6 in Fig. 6): Spherical grain; triporate; isopolar; circular polar view; microrugulate or microechinate; diameter: 25–30 µm.

***Taxodium* sp.** (#7 in Fig. 6): Spherical grain; monoporate; Psilate to scabrate; round pore at exit papilla; papilla length: 3–5 µm; pore diameter: 1–2 µm; diameter: 25–29 µm.

***Ulmus* sp.** (#8 in Fig. 6): Spherical grains; pentaporate; circular amb; oblate; verrucate to scabrate; circular pores; diameter: 30–35 µm.

**Fig. 7:**
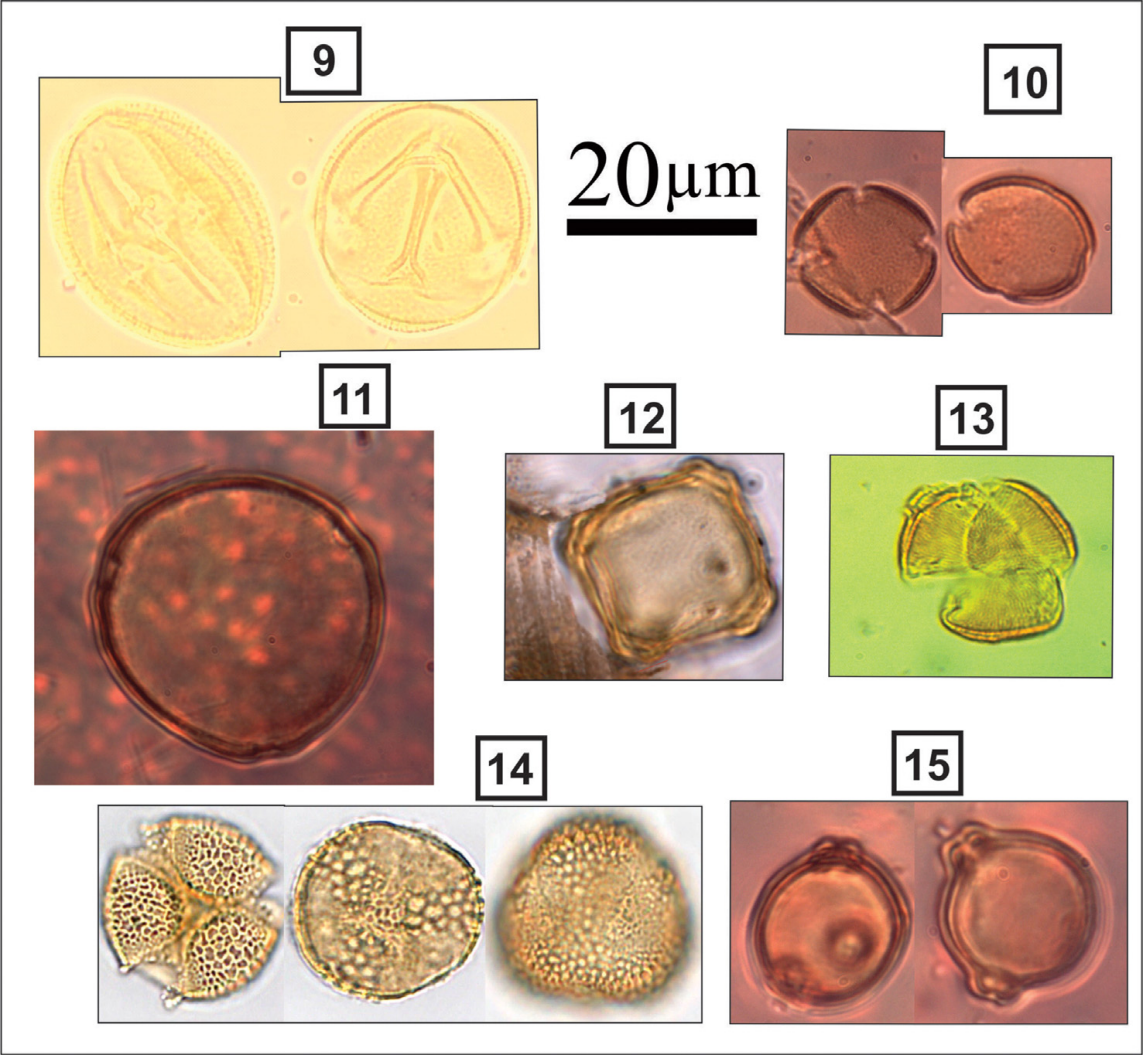
Light micrographs of tree and shrub taxa, including: **9.** Vitaceae; **10.***Fraxinus* sp.; **11.***Carya* sp.; **12**. *Alnus* sp.; **13.***Acer* sp.; **14.***Salix* sp.; **15.***Betula* sp.

**Vitaceae** (#9 in Fig. 7): Subspheroidal to prolate grain; tricolporate; finely reticulate; colpus width: 1–2 µm; round pore; polar axis: 23–28 µm; equatorial axis: 20–26 µm.

***Fraxinus* sp.** (#10 in Fig. 7): Spherical grain; tetracolpate and tricolpate; finely reticulate; isopolar; short colpi; large circular polar view; diameter: 18–20 µm.

***Carya* sp.** (#11 in Fig. 7): Spherical grains; triporate; heteropolar; circular polar view; microechinate; perforate; round and distinct pore; no annulate; diameter: >40 µm.

***Alnus* sp.** (#12 in Fig. 7): Oblate grains; isopolar; radially symmetric; stephanoporate; sexine psilate slightly scabrate appearing as rugulate; amb circular irregular to pentagonal; size: 18-20 µm.

***Acer* sp.** (#13 in Fig. 7): Prolate grain; tricolpate; striate; colpus length: 23–34 µm; polar axis: 29–46 µm; equatorial axis: 30–34 µm.

***Salix* sp.** (#14 in Fig. 7): Subspheroidal grain; tricolporate; reticulate; reticulum finer along colpi and coarser in inter-colpate areas; colpus length: 19–27 µm; oval pore; polar axis: 20–25 µm; equatorial axis: 22–28 µm.

***Betula* sp.** (#15 in Fig. 7): Spherical grains; triporate; vestibulate (separation of endexine); psilate; isopolar; circular polar view; diameter: 20–30 µm.

### 2.6 Identification key (herbaceous taxa)

**Fig. 8:**
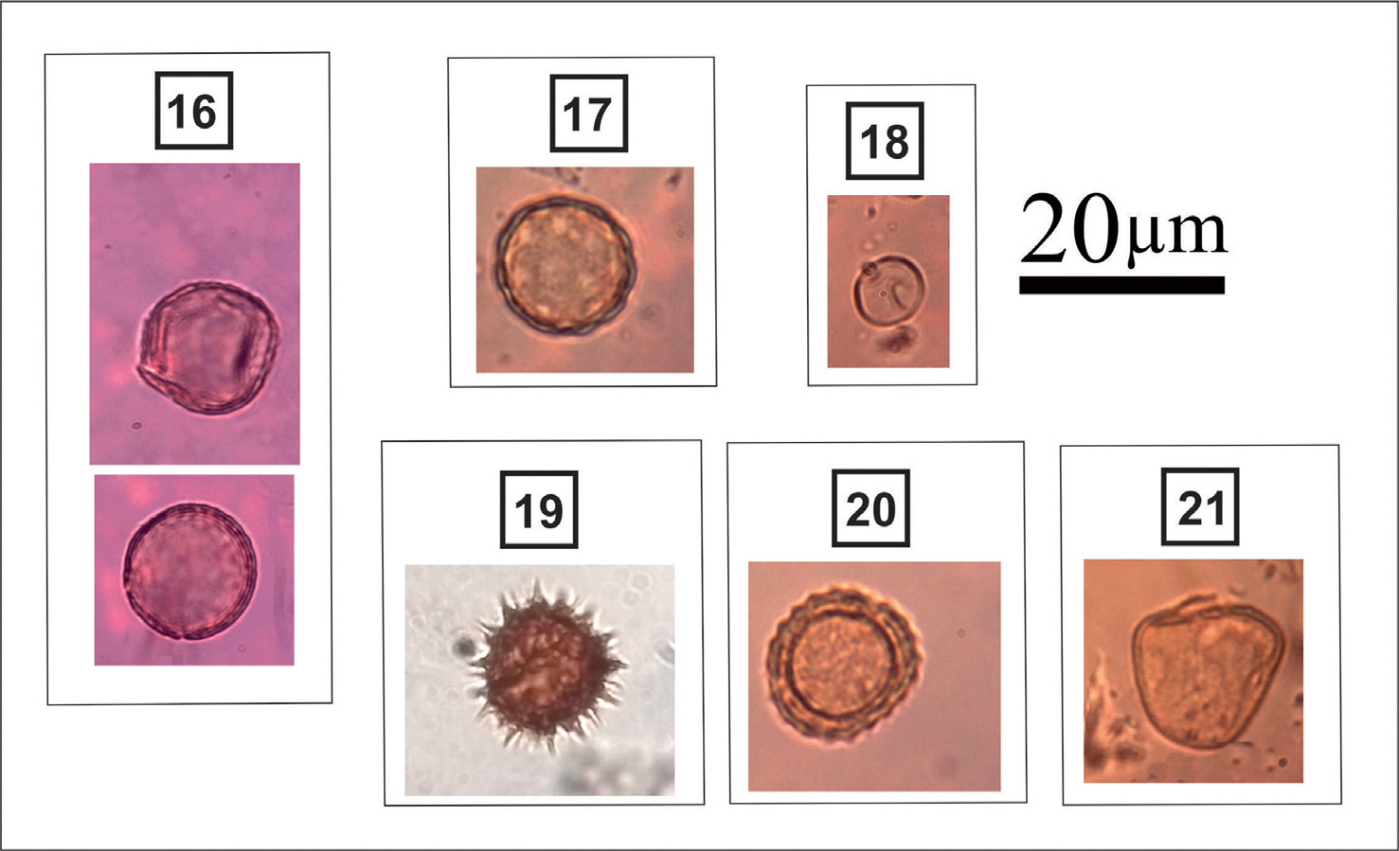
Light micrographs of herbaceous taxa, including: **16.***Plantago* sp.; **17.***Amaranthus* sp.; **18.** Urticaceae; **19.** Asteraceae; **20.***Ambrosia* sp.; **21.***Cyperus* sp.

***Plantago* sp.** (#16 in Fig. 8): Spherical grain; pantoporate; circular amb; scabrate, gemmate, or verrucate; pollen diameter: 15-18 µm.

***Amaranthus* sp.** (#17 in Fig. 8): Spherical grain; periporate; pitted; round pores; pore diameter: 2-4 µm; pollen diameter: 25-30 µm.

**Urticaceae** (#18 in Fig. 8): Spherical grain; diporate; scabrate; round pores; pore diameter: 1-1.3 µm; pollen diameter: 13-16 µm.

**Asteraceae** (#19 in Fig. 8): Subspheroidal grain; tricolporate – tetracolporate – syncolporate; echinate; colpus length: 2–3 µm; pore obscured by sculpture; polar axis: 15–25 µm; equatorial axis: 16–26 µm.

***Ambrosia* sp.** (#20 in Fig. 8): Subspheroidal grain; tricolporate; echinate with short echinae; colpus width: 1–2 µm; pore obscured by sculpture; polar axis: 23–27 µm; equatorial axis: 23–30 µm.

***Cyperus* sp.** (#21 in Fig. 8): Rounded triangular grain (pear-shaped); scabrate; ulcerate; size: 20-28 µm.

**Fig. 9:**
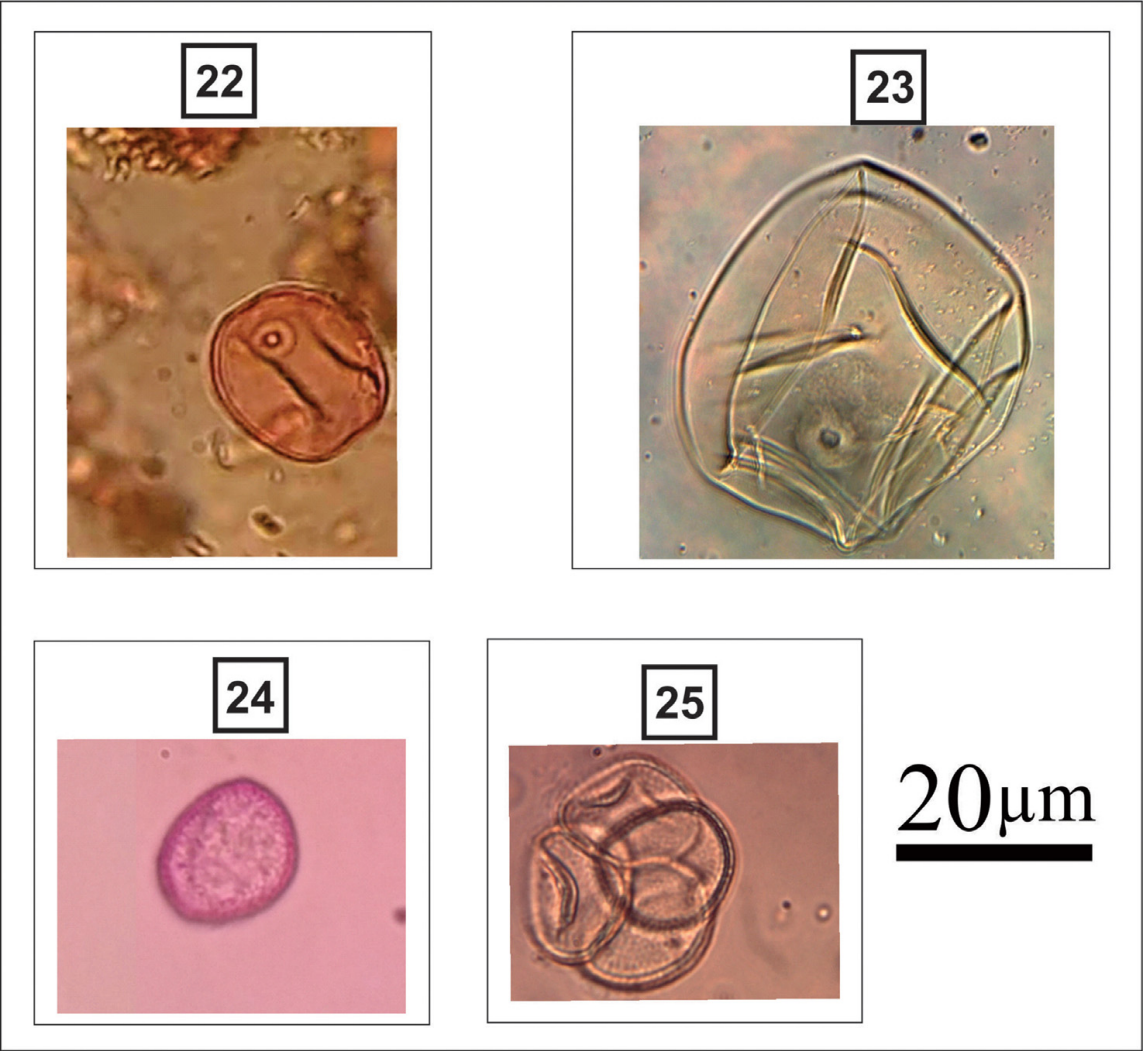
Light micrographs of herbaceous taxa, including: **22.** Poaceae; **23.***Zea mays*; **24**. *Typha angustifolia*; **25.***Typha latifolia*.

**Poaceae** (#22 in Fig. 9):

Spherical grain; Monoporate; round annulate pore; polar diameter: 3–4 µm; psilate to scabrate; size: 30–53 µm.

***Zea mays* (corn)** (#23 in Fig. 9):

Spherical grain; Monoporate; round annulate pore; polar diameter: 8–10 µm; psilate to scabrate; size: 80–125 µm.

***Typha* spp.** (#24, 25 in Fig. 9):

Spherical grain; monad or tetrad; reticulate; monoulcerate; individual size: 20-25 µm.

### 2.7 Identification key (non-pollen palynomorphs) [14]

**Fig. 10:**
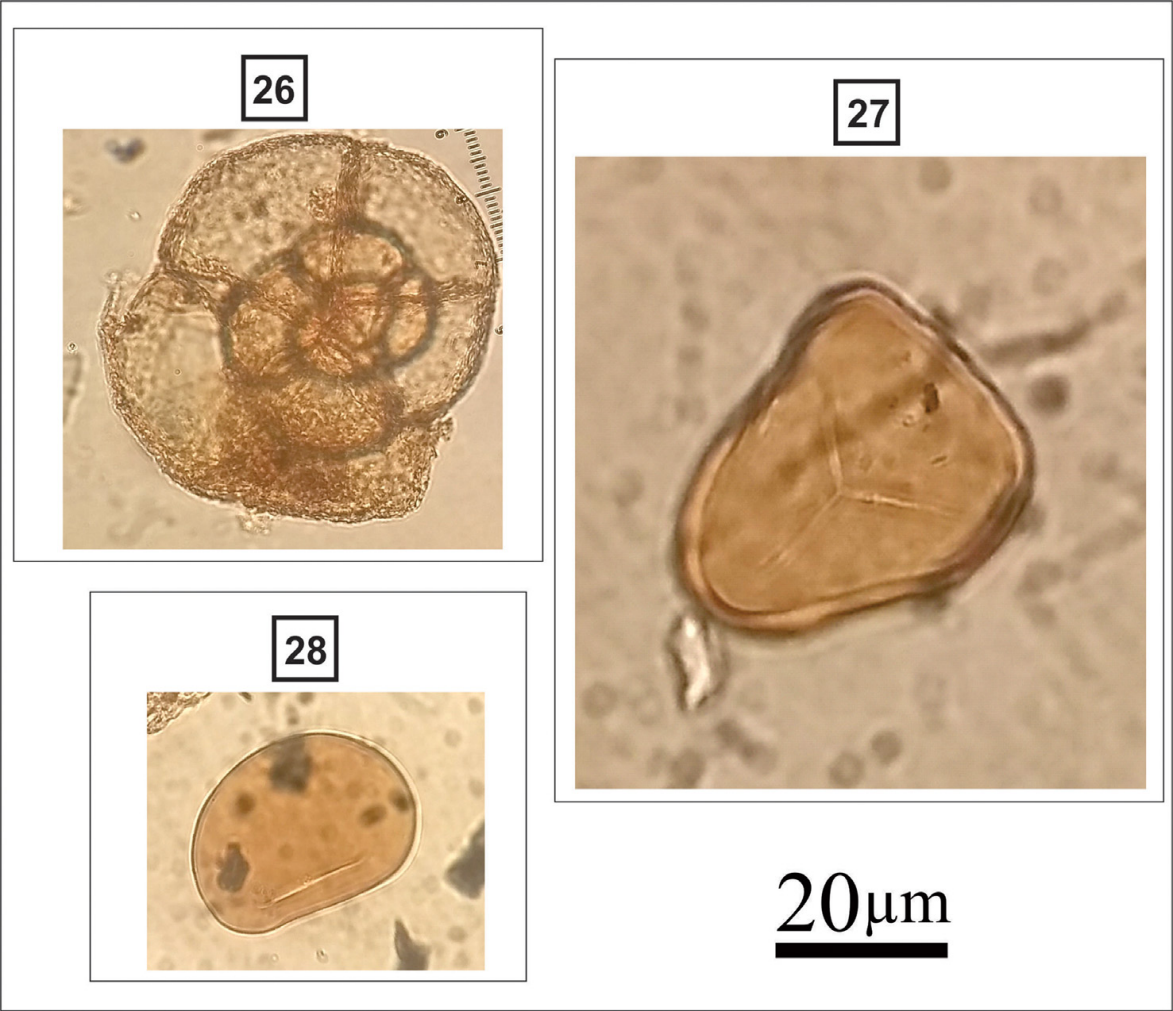
Light micrographs of non-pollen palynomorphs, including: **26.** Microforaminifera; **27.***Acrostichum* sp.; **28**. Monolete spore.

**Microforaminifera (Foraminifera test)** (#26 in Fig. 10):

Microscopic, single-celled organisms that live in wide range of salinity, from freshwater to marine environment. Common species that inhabit the Gulf of Mexico include *Ammonia* sp. and *Elphidium* sp.. Typical size of foraminifera tests ranges from 30 µm to over 1 cm.

***Acrostichum* sp.** (#27 in Fig. 10):

Subtriangular to triangular spore; trichotomosulcate; trilete; scabrate; thick sporoderm; polar axis: 44–55 µm; equatorial axis: 27–32 µm.

**Monolete spore** (#28 in Fig. 10):

Single laesura; with or without perine; kidney bean shape.

## 3 Experimental Design, Materials and Methods

For pollen analysis, cores CK-2 (180 cm; from Atsena Otie Key, 29°7’23.60” N, 83° 2′2.60" W) and SNK-2 (195 cm; from Seahorse Key, 29°5’57.96” N, 83°4’2.22” W) were retrieved in December 2018 by means of a vibra-corer from the fringing mangrove forests on these two islands in Cedar Keys, Florida, U.S.A. The cores were measured, photographed, and wrapped in the field and stored in a cold room (4° C) at the LSU Global Change and Coastal Paleoecology Laboratory. A total of 58 samples consisting of ∼1 cm3 of sediment each were taken from core CK-2 and SNK-2 at a 5-cm interval for palynological analysis. All samples were processed following the standard procedure [15]. One *Lycopodium* tablet (∼20,848 grains) was added to each sample as an exotic marker to calculate the pollen concentration (grains/cm3). A minimum of 300 pollen grains were counted for each sample (except for samples from 130 to 190 cm in core SNK-2 where the pollen concentration is very low) to ensure the results were statistically robust. In addition, foraminifera linings, dinoflagellates, fungal spores, and charcoal fragments (>10 mm in size) were also counted.

For diatom analysis, Core BBS-1 (185 cm; 29°22′10.60′′ N, 94°44′0.20′′ W) was retrieved in December 2018 using a vibra-corer from the black mangrove stands on the flat. The core was pushed in until refusal to capture the most complete depositional history possible. Twenty-nine samples consisting of ∼1 cm^3^ of sediment were collected at 2-5 cm intervals for diatom analysis, following the standard procedure and treatment described in Tomas et al. [16] and Wachnicka et al. [17]. Diatom identification was based on microstructural analysis of the silicified cell wall, such as pore size, external layer, volume of diatoms, and shapes. In order to yield a statistically meaningful relationship, at least 500 valves were counted for each sample. TILIA and TILIAGRAF software were used for calculation and plotting the pollen diagram [18]. CONISS was used for cluster analysis of the pollen data [19].

## Ethics Statements

This study does not involve any human subjects or animal experiments.

## CRediT Author Statement

**Erika Rodrigues:** Data curation. **Kam-biu Liu:** Supervision. **Paulo Eduardo De Oliveira:** Resources **Beatriz L Figueiredo:** Visualization **Qiang Yao:** Methodology, writing- Original draft preparation.

## Declaration of Competing Interest

The authors declare that they have no known competing financial interests or personal relationships that could have appeared to influence the work reported in this paper.

## Data Availability

Pollen dataset from Cedar Keys, Florida, USA (Original data) (Mendeley Data). Original diatom counts of core BBS-1 from Galveston Bay, Texas, USA (Original data) (Mendeley Data).
